# MOMAST^®^ Downregulates AQP3 Expression and Function in Human Colon Cells

**DOI:** 10.3390/antiox14010026

**Published:** 2024-12-28

**Authors:** Ines Angelini, Mariangela Centrone, Giusy Rita Caponio, Annarita Di Mise, Andrea Gerbino, Marianna Ranieri, Giovanna Valenti, Grazia Tamma

**Affiliations:** 1 Department of Biosciences, Biotechnologies, and Environment, University of Bari “Aldo Moro”, 70125 Bari, Italy; ines.angelini@uniba.it (I.A.); mariangela.centrone@uniba.it (M.C.); giusy.caponio@uniba.it (G.R.C.); andrea.gerbino@uniba.it (A.G.); marianna.ranieri@uniba.it (M.R.); giovanna.valenti@uniba.it (G.V.); 2 Department of Biotechnology and Biosciences, University of Milan-Bicocca, 20126 Milan, Italy; annarita.dimise@unimib.it

**Keywords:** AQP3, MOMAST^®^, EMT, vimentin, colon cells, polyphenols, waste and by-products

## Abstract

The water channel AQP3 is an aquaglyceroporin expressed in villus epithelial cells, and it plays a role in water transport across human colonic surface cells. Beyond water, AQP3 can mediate glycerol and H_2_O_2_ transport. Abnormal expression and function of AQP3 have been found in various diseases often characterized by altered cell growth and proliferation. Here, the beneficial effects of MOMAST^®^ have been evaluated. MOMAST^®^ is an antioxidant-patented natural phenolic complex obtained from olive wastewater (OWW) of the Coratina cultivar. Treatment of human colon HCT8 cells with MOMAST^®^ reduced cell viability. Confocal studies and Western Blotting analysis demonstrated that treatment with MOMAST^®^ significantly decreased the staining and the expression of AQP3. Importantly, functional studies revealed that the reduction of AQP3 abundance correlates with a significant decrease in glycerol and H_2_O_2_ uptake. Indeed, the H_2_O_2_ transport was partially but significantly reduced in the presence of MOMAST^®^ or DFP00173, a selective inhibitor of AQP3. In addition, the MOMAST^®^-induced AQP3 decrease was associated with reduced epithelial-mesenchymal transition (EMT)-related proteins such as vimentin and β-catenin. Together, these findings propose MOMAST^®^ as a potential adjuvant in colon diseases associated with abnormal cell growth by targeting AQP3.

## 1. Introduction

Aquaporins (AQPs) are water-mediated channels expressed in the cell membranes [1]. Mammalian cells express more than 13 AQPs that can be classified into three main groups including classical AQPs (AQP0, AQP1, AQP2, AQP4, AQP5, AQP6, and AQP8), aquaglyceroporins (AQP3, AQP7, AQP9, and AQP10), and unorthodox aquaporins (AQP11 and AQP12) [2]. Within AQPs, some of them (AQP3, AQP5, AQP6, AQP7, AQP8, AQP9, and AQP11) are known to be peroxiporins as they also mediate the transport of hydrogen peroxide (H_2_O_2_) [1], thereby modulating the intracellular redox state. Of these, AQP3 is highly expressed in the gastrointestinal tube [3], where it plays paramount roles in controlling the transport of water, glycerol, and/or H_2_O_2_ [1,2,4]. Additionally, AQP3 has been involved in numerous intracellular responses including cell proliferation and epithelial-mesenchymal transition (EMT) [5]. Based on these findings, emerging evidence revealed that abnormal expression and functions of AQP3 are related to the onset of numerous diseases including diarrhea, constipation, intestinal oxidative stress, and cancer. Also, RNA-Seq analysis revealed that AQP3 gene expression increased in colon cancer compared to the normal colonic mucosa [6]. Physiologically, the gut epithelium represents a pivotal site involved in water transport through paracellular or transcellular pathways. Nevertheless, due to the tight cell junction complex, osmotically driven water movement occurs mainly through the transcellular route via AQPs. In line, constipation is associated with an abnormal expression of colonic AQP3 [7]. Conversely, selective inhibition of AQP3 increases the fecal water content, thereby leading to diarrhea [8]. Importantly, in deficient AQP3 transgenic mice, a significant decrease in enterocyte proliferation and migration has been described [9]. Accordingly, the reduction in the expression and function of AQP3 induced by DDAVP is associated with a decrease in cell viability. This may be due to reduced intracellular glycerol availability and energy production, which are essential for cell proliferation processes [6]. On the other hand, abnormal cell proliferation can be the result of excessive intracellular levels of reactive oxygen species (ROS) derived from cellular aerobic metabolism and impaired neutralization of ROS systems. Abnormal accumulation of reactive species (ROS; RNS) can cause oxidative stress that contributes to the initiation and progression of several disorders such as diabetes, obesity, and cancer [10]. In fact, ROS can act as intracellular signaling molecules that promote mitogenic pathways [11]. Thus, ROS can stimulate oncogenesis by interfering with intracellular processes that control cell proliferation and death. Also, elevated ROS levels can lead to angiogenesis, immunological tolerance, and epithelial-mesenchymal transition (EMT) [12]. Importantly, under specific conditions, phenolic acids may also exert pro-oxidant activity due to H_2_O_2_ generation that derives from phenolic acid autoxidation. Thus, H_2_O_2_ transport through peroxiporins stimulates oxidative-dependent pathways leading to oncogenesis [13]. Consistent with this, the inhibition of AQP3 function, by using the selective inhibitor DFP00173, has been shown to significantly reduce intracellular transport of H_2_O_2_ [13]. Moreover, in an in vivo model of colitis, the protective effect of quercetin correlates with a relevant reduction of AQP3 expression level and a lower AQP3-dependent H_2_O_2_ transport capability through the plasma membrane [14]. As a result, AQP3 has been proposed as a key target for nutrients and bioactive compounds [15] that are involved in the improvement of gut health. Therefore, the major aim of this study was to investigate the biological action and the beneficial effects of MOMAST^®^ on AQP3 expression and function using the human ileocecal adenocarcinoma HCT8 cells as an experimental model. MOMAST^®^ is a patented polyphenolic complex derived from olive oil mill wastewater of the Coratina cultivar that is enriched in several natural phenols including hydroxytyrosol, tyrosol, oleuropein, and verbascoside [16]. Previous findings have demonstrated that MOMAST^®^ exhibits radical scavenging and ferric reducing capacities [17,18]. In this respect, MOMAST^®^ has been biochemically and functionally characterized by Recinella [19]. Specifically, in vitro and in vivo studies have indicated that MOMAST^®^ has anti-pain actions, exerts significant anti-inflammatory actions [19], and may be proposed as a food supplement in patients with irritable bowel syndrome (IBS) [20]. Additional evidence revealed that MOMAST^®^ ameliorates the plasmatic lipid profile by lowering cholesterol levels and improving ROS metabolism [16,17]. Here, we found that increasing concentration of MOMAST^®^ correlates with a significant reduction in cell viability, AQP3 intracellular expression, and function in terms of glycerol and H_2_O_2_ transport.

## 2. Materials and Methods

### 2.1. Chemicals and Reagents

Cell culture media and FBS (fetal bovine serum) were from GIBCO (Thermo Fisher Scientific™, Waltham, MA, USA).

MOMAST^®^ [16,20,21] was a generous gift from the Bioenutra S.R.L. company (Bioenutra S.R.L., Ginosa TA, Italy), and it has been biochemically characterized by Recinella et al. [19].

Calcein-AM was from Invitrogen (Thermo Fisher Scientific™, Waltham, MA, USA).

TransFectin™ Lipid Reagent and Clarity Western ECL Substrates were bought from Bio-Rad (Bio-Rad Laboratories, Inc., Hercules, CA, USA).

### 2.2. Antibodies

To detect the total amount of AQP3 [6], the rabbit polyclonal antibody was bought from Invitrogen (Cat# PA5-53257, Thermo Fisher Scientific™, Waltham, MA, USA). The rabbit polyclonal antibody against vimentin was purchased from Proteintech (Cat# 10366-1-AP, Proteintech Group, Inc., Rosemont, IL, USA).

The mouse monoclonal antibody against β-catenin was obtained from Santa Cruz Biotechnology (sc-7963, Santa Cruz Biotechnology, Inc., Dallas, TX, USA).

The E-Cadherin antibody was from Millipore Merck (Cat# 07-697, Merck KGaA, Darmstadt, Germany). The Anti-Rabbit IgG (whole molecule) and the Anti-Mouse IgG (whole molecule) secondary antibodies were obtained from Sigma-Aldrich (Merck KGaA, Darmstadt, Germany).

Secondary donkey anti-rabbit antibodies coupled to Alexa-488 were purchased from Thermo Fisher Scientific (Thermo Fisher Scientific™, Waltham, MA, USA).

### 2.3. The Flowchart of the Study

**Scheme 1:**
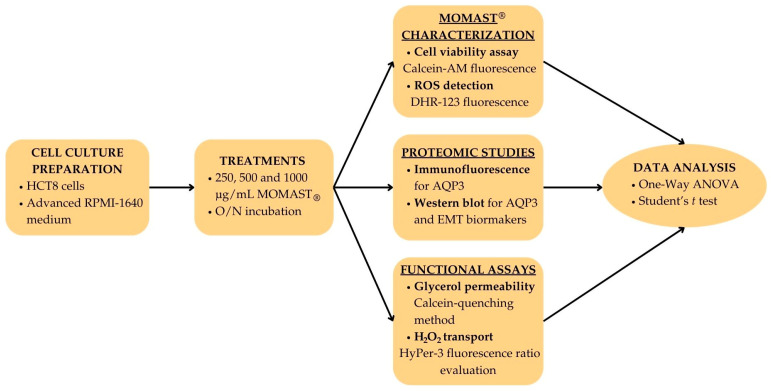
Flowchart showing the experimental protocol applied in the present study.

In this paragraph, the proposed methods applied to evaluate the biological effects of MOMAST^®^ on AQP3 expression and function are presented in detail. Specifically, Scheme 1 graphically shows the experimental protocol and procedures including cell culturing, biochemical and biophysical analysis, and statistical methods.

### 2.4. Cell Culture and Treatments

Human HCT8 cells, an ileocecal adenocarcinoma cell line, were grown, as previously described [6], in Advanced RPMI-1640 supplemented with 10% FBS, 100 i.u.·mL^−1^ penicillin, 100 µg·mL^−1^ streptomycin at 37 °C in 5% CO_2_. Cells were grown at 80–90% confluence. Cells were left under basal condition (untreated) or incubated with MOMAST^®^ at 50, 100, 250, 500, or 1000 µg/mL overnight.

### 2.5. Cell Viability Assay

Cell viability was evaluated using Calcein-AM as already described [22]. After treatments, cells were incubated with Calcein-AM (1 µM) at 37 °C for 45 min. Only in viable cells, the intracellular esterases cut the acetoxymethyl ester (AM) group, and the green fluorescent dye accumulates. Then, the fluorescence emission signal was measured using the FLUOstar Omega Microplate Reader (BMG Labtech, Ortenberg, Germany). As an internal positive control (CTR+), cells were incubated with ethanol (90%) for 1 min.

### 2.6. Reactive Oxygen Species (ROS) Detection

ROS were measured as already shown [23]. After treatments, cells were incubated with 10 μM dihydrorhodamine-123 at 37 °C for 30 min and then recovered in a complete medium for 30 min. Then, to obtain the intracellular content, the RIPA lysis buffer (150 mM NaCl, 10 mM Tris-HCl pH 7.2, 0.1% SDS, 1% Triton X-100, 1% sodium deoxycholate, and 5 mM EDTA pH 8) was used. After centrifugations (12,000× *g* for 10 min at 4 °C), the supernatants were collected and used for ROS levels measurements. The fluorescence emission signal was detected through the FLUOstar Omega Microplate Reader (BMG Labtech, Ortenberg, Germany) and then normalized to the total protein content. As an internal positive control, cells were treated with 2 mM tert-butyl hydroperoxide (tBHP) for 30 min.

### 2.7. Immunofluorescence

For the immunofluorescence experiments, HCT8 cells were seeded on 12 mm Ø glass coverslips. At complete confluence, cells were fixed in 4% paraformaldehyde solution for 20 min and washed three times with phosphate-buffered saline (PBS) with calcium and magnesium. Cells were transiently permeabilized with 0.1% Triton X-100 in PBS for 5 min, and the unspecific binding sites were blocked through the incubation with 1% bovine serum albumin (BSA) in PBS. Then, the AQP3 specific primary antibodies were used overnight at 4 °C followed by the incubation with the 488 Alexa Fluor-conjugated secondary antibodies for 1 h at 37 °C. After washings, glass coverslips were mounted onto glass slides with Mowiol^®^ mounting medium. Images were acquired with a confocal laser-scanning fluorescence microscope Leica TCS SP2 (Leica Microsystems, Heerbrugg, Switzerland).

### 2.8. Glycerol Permeability Assay

Glycerol permeability in HCT8 cells was measured taking advantage of the Calcein-AM self-quenching capability, as previously described [6,24,25].

Cells were seeded on 25 mm Ø glass coverslips, and at a confluence of approximately 70%, they were exposed to treatments. Samples were loaded with 12 μM Calcein-AM in Advanced RPMI 1640 for 45 min at 37 °C, and then the coverslips were mounted in a perfusion chamber exposed to an isotonic solution (137 mM NaCl, 5.4 mM KCl, 0.5 mM MgCl_2_, 1.3 mM CaCl_2_, 4.2 mM NaHCO_3_, 0.4 mM KH_2_PO_4_, 3 mM Na_2_HPO_4_, 10 mM Hepes sulfonic acid, 10 mM glucose, pH 7.4). Single-cell changes in fluorescence were recorded by using an inverted fluorescence microscope (Nikon ECLIPSE TE2000-S, Tokyo, Japan) equipped with a cooled CCD camera controlled by the Metafluor 4.6 software (Molecular Devices, MDS Analytical Technologies, Toronto, ON, Canada).

A 100 mOsm/L osmotic gradient was generated by adding glycerol to the isotonic solution. Time course fluorescence data were recorded every 5 s in both the shrinking phase, due to the osmotic water exit, and the later swelling phase, due to the osmotic influx of water following glycerol entry along its gradient.

For the data analysis, the slope of the cell swelling phase, considered as time constant, was fit to a linear function using GraphPad Software (San Diego, CA, USA). Under such conditions, the calculated time constant is considered an index that reflects the membrane glycerol permeability.

### 2.9. HyPer-3 Imaging

The HyPer-3 is a genetically encoded fluorescent H_2_O_2_ sensor used for the real-time imaging of intracellular H_2_O_2_ [26]. To evaluate changes in cytosolic hydrogen peroxide levels, HCT8 cells were seeded on 25 mm Ø glass coverslips. Cells were transiently transfected with 3 μg pC1-HyPer-3 using 12 μL of TransFectin™ Lipid Reagent (Bio-Rad Laboratories, Inc., Hercules, CA, USA). HyPer-3 was a gift from Vsevolod Belousov (Addgene plasmid # 42131; http://n2t.net/addgene:42131 accessed on 24 November 2024; RRID: Addgene_42131).

After transfection (24 h), coverslips were mounted in an open perfusion chamber and images were acquired using an inverted Eclipse TE2000 microscope (Nikon, Shinagawa, Tokyo, Japan) equipped for single-cell fluorescence evaluation and image analysis. Cells were then exposed to solutions with increasing concentrations of H_2_O_2_ (25, 50, 100 µM). Cells expressing HyPer-3 were illuminated every 5 s at 420 and 500 nm through a 40× oil immersion objective (numerical aperture = 1.30). The oxidation of the sensor alters the fluorescence response after ratiometric excitation at 420 and 500 nm, respectively, thereby increasing the fluorescence ratio (F500/F420) [27]. The emitted fluorescence was passed through a dichroic mirror, filtered at 515 nm (Omega Optical, Brattleboro, VT, USA), and captured by a cooled CCD CoolSNAP HQ camera (Photometrics, Tucson, AZ, USA). Fluorescence ratio measurements were carried out using the MetaFluor Fluorescence Ratio Imaging Software (Version 7.7.3.0, Molecular Devices, San Jose, CA, USA).

### 2.10. Gel Electrophoresis and Immunoblotting

According to the manufacturer’s instructions, SDS-PAGE was performed on 12% polyacrylamide gels (Bio-Rad Laboratories, Inc., Hercules, CA, USA). Protein bands were transferred onto the PVDF Transfer membrane (Thermo Fisher Scientific, Waltham, MA, USA) and subjected to immunoblotting using specific antibodies [6]. To improve the sensitivity and specificity of the bands, the EveryBlot blocking buffer was applied (Bio-Rad Laboratories, Inc., Hercules, CA, USA). Proteins were detected using Clarity Western ECL Substrate with the ChemiDoc System gels (Bio-Rad Laboratories, Inc., Hercules, CA, USA). Specific bands were normalized to total protein content by applying the Stain-free technology gels. Densitometric analysis of the bands was performed using Image Lab software (Bio-Rad Laboratories, Inc., Hercules, CA, USA).

### 2.11. Statistical Analysis

GraphPad Prism version 8.0.1 (GraphPad Software, San Diego, CA, USA) was used for the statistical analysis and graphs representation.

The one-way analysis of variance (ANOVA) followed by Dunnett’s multiple comparisons test was performed. When applicable, the Student’s *t* test was also used.

Values are shown as means ± Standard Error Means (S.E.M.). The difference of *p* < 0.05 was considered statistically significant.

## 3. Results

### 3.1. Characterization of MOMAST^®^ in HCT8 Cells

**Figure 1:**
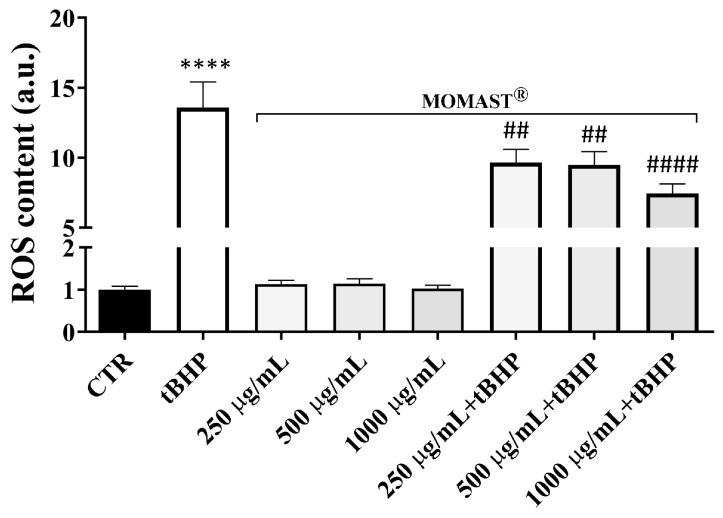
ROS content in HCT8 cells exposed to MOMAST^®^. ROS content was measured using dihydrorhodamine-123 fluorescence in cells left under basal conditions or treated with MOMAST^®^ as described in Methods. As a positive control, cells were treated with the oxidant *tert*-Butyl hydroperoxide (tBHP). Untreated cells (CTR) are a blank control. Data are shown as means ± Standard Error Means (S.E.M.) of six independent experiments and analyzed with one-way ANOVA followed by Dunnett’s multiple comparisons test (**** *p* < 0.0001 vs. CTR; **^##^** *p* < 0.01 vs. tBHP; **^####^** *p* < 0.0001 vs. tBHP).

MOMAST^®^ displays several protective actions on inflammatory and oxidative stress signal transduction pathways [17,20,21]. Here, the antioxidant effect of MOMAST^®^ was evaluated in HCT8 cells. Cells were treated at increasing concentrations with MOMAST^®^ (250, 500, and 1000 µg/mL overnight) in the absence or in the presence of tBHP which was used as an internal positive control (Figure 1). Compared to the positive control, exposure to MOMAST^®^ resulted in a significant reduction in tBHP-induced ROS levels indicating its antioxidant capacity (Supplementary Materials, Table S1). Treatment with MOMAST^®^ alone did not alter the intracellular ROS levels compared to untreated cells (CTR).

**Figure 2:**
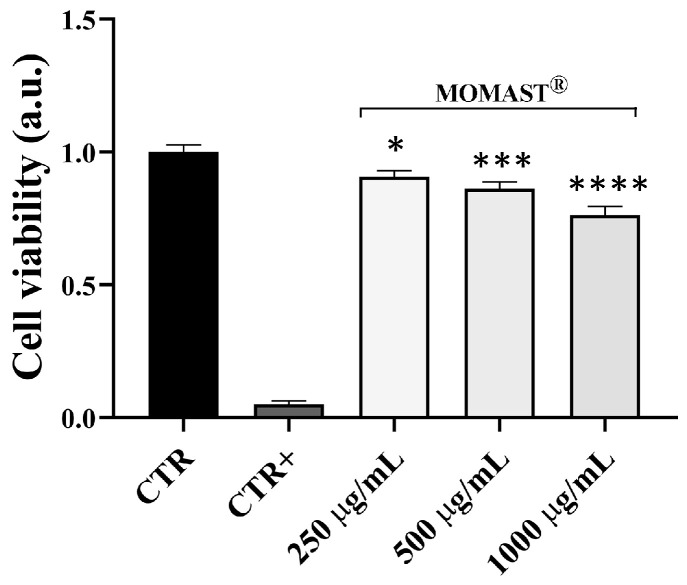
Cell viability in HCT8 cells exposed to MOMAST^®^. Cells were left under basal conditions or exposed to increasing concentrations (250, 500, and 1000 µg/mL) of MOMAST^®^ as described in Methods. As a positive internal control, cells were treated with 90% ethanol for 1 min (CTR+). Data are shown as means ± Standard Error Means (S.E.M.) of seven independent experiments and analyzed with one-way ANOVA followed by Dunnett’s multiple comparisons test (* *p* < 0.05 vs. CTR; *** *p* < 0.001 vs. CTR; **** *p* < 0.0001 vs. CTR).

Importantly, several phenols, with antioxidant activity, may also modulate cell viability [28]. Therefore, the potential effect of MOMAST^®^ on HCT8 cell viability was investigated using the fluorophore Calcein-AM (see Methods for details). The Calcein-AM-based assay showed that MOMAST^®^ at 250, 500, and 1000 µg/mL significantly reduced cell viability (Figure 2). At lower concentrations (50 and 100 µg/mL), MOMAST^®^ did not alter cell viability (CTR: 1.00 ± 0.03; CTR+: 0.05 ± 0.01; MOMAST^®^ 50 µg/mL: 0.96 ± 0.02; MOMAST^®^ 100 µg/mL: 0.95 ± 0.03; MOMAST^®^ 250 µg/mL: 0.91 ± 0.02 *; MOMAST^®^ 500 µg/mL: 0.86 ± 0.02 ***; MOMAST^®^ 1000 µg/mL: 0.76 ± 0.03 ****; * *p* < 0.05 vs. CTR, *** *p* < 0.001 vs. CTR, **** *p* < 0.0001 vs. CTR, *n* = seven independent experiments).

### 3.2. MOMAST^®^ Action on AQP3 Expression and Function

**Figure 3:**
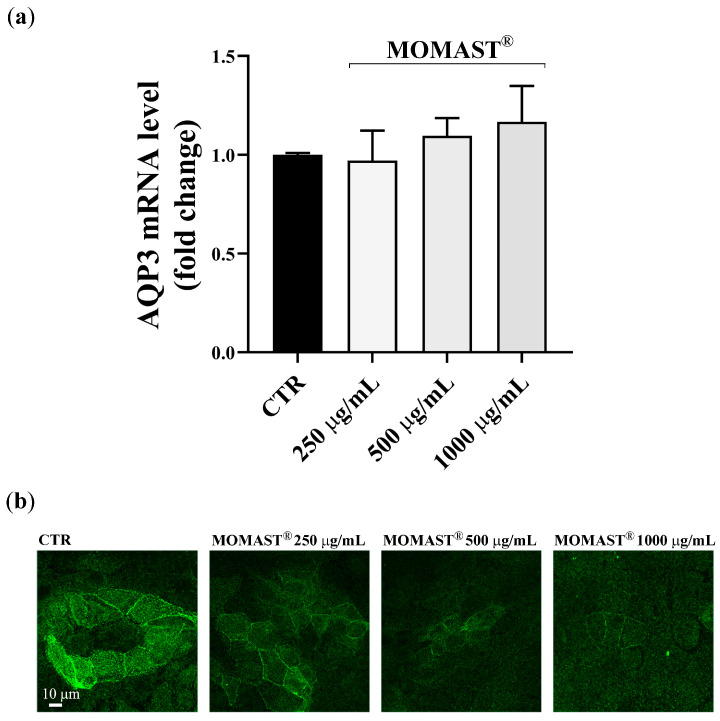
(**a**) Real-time PCR analysis of AQP3 mRNA expression levels. MOMAST^®^ treatment did not alter the AQP3 transcript levels in HCT8 cells. Data are shown as means ± Standard Error Means (S.E.M.) of three independent experiments and analyzed with one-way ANOVA followed by Dunnett’s multiple comparisons test. (**b**) Confocal immunofluorescence visualization of AQP3 in HCT8 cells. Cells were left under basal conditions or exposed to increasing concentrations (250, 500, and 1000 µg/mL) of MOMAST^®^ as described in Methods. Confocal images show a slight but relevant reduction in AQP3 fluorescence signal.

Previous studies using HCT8 cells as an experimental model showed that the reduction in cell viability was associated with a significant decrease in the expression of the aquaglyceroporin AQP3 [6]. Real-time PCR experiments (Figure 3a) were therefore performed to test the possible effect of MOMAST^®^ on AQP3 mRNA expression. Cells were left untreated or incubated with MOMAST^®^ at 250, 500, and 1000 µg/mL overnight. Data showed that MOMAST^®^ did not alter the AQP3 mRNA expression at the concentration used in this study (Figure 3a, CTR: 1.00 ± 0.01; MOMAST^®^ 250 µg/mL: 0.97 ± 0.15; MOMAST^®^ 500 µg/mL: 1.10 ± 0.09; MOMAST^®^ 1000 µg/mL: 1.17 ± 0.18; *n* = three independent experiments). Also, immunofluorescence revealed that AQP3 is mainly expressed in the plasma membranes (Figure 3b). However, a slight but relevant reduction of the AQP3 expression was found in HCT8 cells treated with MOMAST^®^.

**Figure 4:**
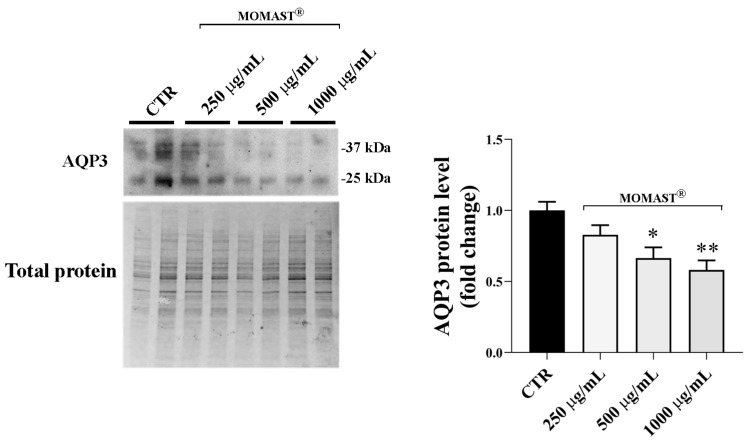
Effect of MOMAST^®^ on AQP3 protein expression. Treatments with MOMAST^®^ at increasing concentrations induced a significant reduction in the AQP3 protein expression. Data are shown as means ± Standard Error Means (S.E.M.) of 5 independent experiments and analyzed by one-way ANOVA followed by Dunnett’s multiple comparisons test (* *p* < 0.05 vs. CTR; ** *p* < 0.01 vs. CTR).

Western Blotting studies were therefore performed to evaluate the possible action of MOMAST^®^ on AQP3 protein expression (Figure 4). To this end, HCT8 cells were left untreated or incubated as previously mentioned. Western blotting (left panel) and densitometric analysis (right panel) showed that treatments with MOMAST^®^ significantly reduced the AQP3 protein expression compared to the untreated condition (Figure 4; CTR: 1.00 ± 0.06; MOMAST^®^ 250 µg/mL: 0.83 ± 0.07; MOMAST^®^ 500 µg/mL: 0.66 ± 0.07 **; MOMAST^®^ 1000 µg/mL: 0.58 ± 0.07 ***; ** *p* < 0.01 vs. CTR, *** *p* < 0.001 vs. CTR, *n* = five independent experiments).

**Figure 5:**
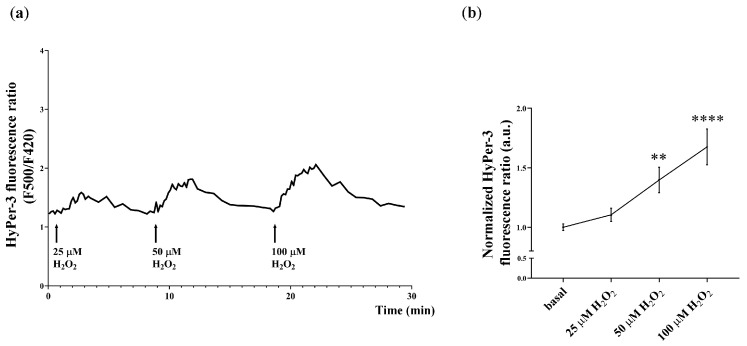
Evaluation of the HyPer-3 sensitivity to H_2_O_2_ exposure by analyzing the HyPer-3 fluorescence ratio. (**a**) A representative trace showing the HyPer-3 fluorescence ratio in transfected HCT8 cells stimulated with external H_2_O_2_ at increasing concentrations (25, 50, and 100 μM). (**b**) A significant increase in normalized HyPer-3 fluorescence ratio intensity was recorded at 50 and 100 μM H_2_O_2_ external stimuli. Data are shown as means ± Standard Error Means (S.E.M.) of six independent experiments and analyzed with one-way ANOVA followed by Dunnett’s multiple comparisons test (** *p* < 0.01 vs. basal; **** *p* < 0.0001 vs. basal).

To further investigate the effect of MOMAST^®^ on AQP3 function, H_2_O_2_ transport was evaluated. Cells were transiently expressing the H_2_O_2_ fluorescent probe, HyPer-3, and were exposed to increasing concentrations of external H_2_O_2_ (25, 50, and 100 µM). Figure 5a shows a representative time course revealing that changes in HyPer-3 fluorescence responses were observed with increasing external H_2_O_2_. In particular, there was a significant peak fluorescence response following treatment with external H_2_O_2_ at 50 or 100 µM. (Figure 5b; basal: 1 ± 0.03; 25 µM H_2_O_2_: 1.10 ± 0.05; 50 µM H_2_O_2_: 1.40 ± 0.11 **; 100 µM H_2_O_2_: 1.68 ± 0.15 ****; ** *p* < 0.01 vs. basal, **** *p* < 0.0001 vs. basal, *n* = six independent experiments).

**Figure 6:**
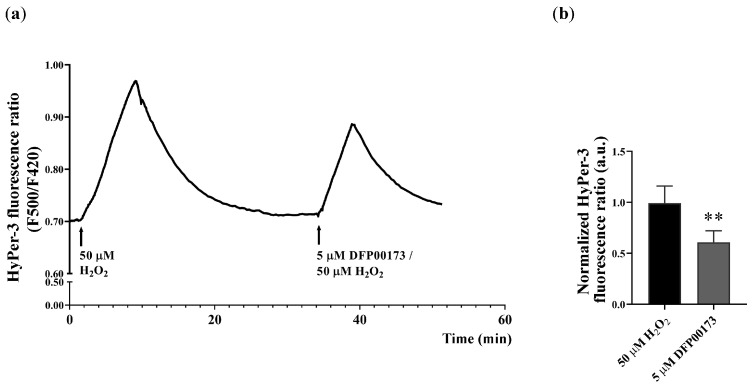
Evaluation of the HyPer-3 sensitivity to H_2_O_2_ exposure by analyzing the HyPer-3 fluorescence ratio in the absence or in the presence of the AQP3 inhibitor, DFP00173. (**a**) A representative trace showing the HyPer-3 fluorescence ratio in HCT8 cells stimulated with external 50 μM H_2_O_2_ in the absence and the presence of 5 μM DFP00173. (**b**) The histogram shows the normalized HyPer-3 fluorescence ratio. The normalized HyPer-3 fluorescence ratio was measured by calculating the fluorescence ratio at the peak compared to the starting fluorescence values. Data are shown as means ± Standard Error Means (S.E.M.) of five independent experiments and analyzed by paired *t* test (** *p* < 0.01 vs. 50 µM H_2_O_2_).

Interestingly, compared to the peak fluorescence response induced by external H_2_O_2_ at 50 µM, selective inhibition of AQP3 using 5 µM DFP00173 reduced the peak fluorescence signal response induced by external H_2_O_2_ at 50 µM, which is consistent with a decrease in H_2_O_2_ transport (DFP000173/50 µM H_2_O_2_: 1.00 ± 0.17 vs. 50 µM H_2_O_2_: 0.61 ± 0.11; ** *p* < 0.01, *n* = 35). This result likely indicates that AQP3 contributes to and mediates H_2_O_2_ uptake (Figure 6) in HCT8 cells.

**Figure 7:**
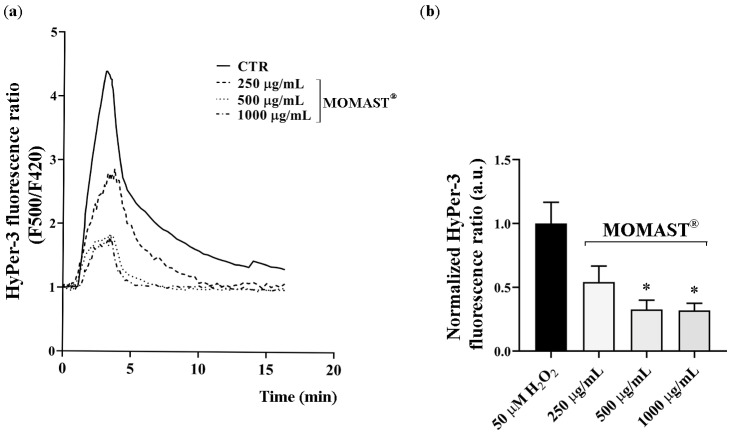
Live-cell imaging of changes in HyPer-3 fluorescence in HCT8 cells. (**a**) Representative traces showing the HyPer-3 fluorescence ratio in HCT8 cells treated with MOMAST^®^. (**b**) The histogram shows the normalized HyPer-3 fluorescence ratio where the normalized HyPer-3 ratio was calculated by evaluating the fluorescence ratio at the peak compared to the starting fluorescence values. Data are shown as means ± Standard Error Means (S.E.M.) of five independent experiments and analyzed by one-way ANOVA followed by Dunnett’s multiple comparisons test (* *p* < 0.05 vs. 50 µM H_2_O_2_).

Cells were treated as described above to evaluate the MOMAST^®^ action on H_2_O_2_ transport ability. Interestingly, exposure to MOMAST^®^ (Figure 7) significantly reduced the H_2_O_2_ uptake induced by external H_2_O_2_ at 50 µM.

**Figure 8:**
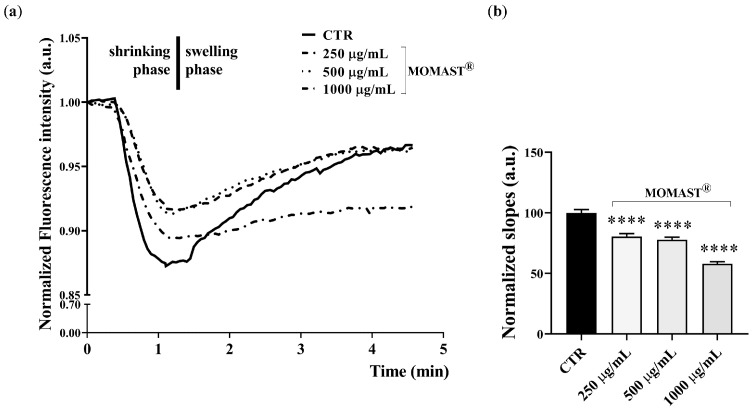
Effect of MOMAST^®^ on glycerol permeability. (**a**) Representative time courses of cell shrinking (water exit) followed by cell swelling indicative of the osmotic influx of water promoted by glycerol entry along its gradient (Δ 100 mOsm/L). (**b**) A bar plot showing the means ± Standard Error Means (S.E.M.) values of the cell swelling time constants (slope) reflecting glycerol entry into the cells. Data are obtained from 91 different measurements of 4 independent experiments. A one-way ANOVA and Dunnett’s Multiple Comparison test were performed (**** *p* < 0.0001 vs. CTR).

In addition, functional studies were performed using a calcein-based assay (Figure 8) to determine whether MOMAST^®^ also affected AQP3-mediated glycerol transport. Compared to untreated cells, stimulation with MOMAST^®^ at 250, 500, and 1000 µg/mL overnight significantly reduced the temporal osmotic response generated using a glycerol hyperosmotic solution likely indicating that the reduction in AQP3 protein expression corresponds to a decrease in the intracellular glycerol uptake. However, to test whether MOMAST^®^ interferes with the AQP3-dependent glycerol proprieties, HCT8 cells were treated with 5 µM DFP00173 for 45 min, a selective inhibitor of AQP3, in the absence or the presence of MOMAST^®^ (Supplementary Materials, Figure S1). Treatment with DFP00173 reduced the basal AQP3-mediated glycerol transport in comparison to untreated cells. In the presence of MOMAST^®^, exposure to DFP00173 further reduced intracellular glycerol uptake.

### 3.3. MOMAST^®^ Action on EMT Biomarker Proteins

**Figure 9:**
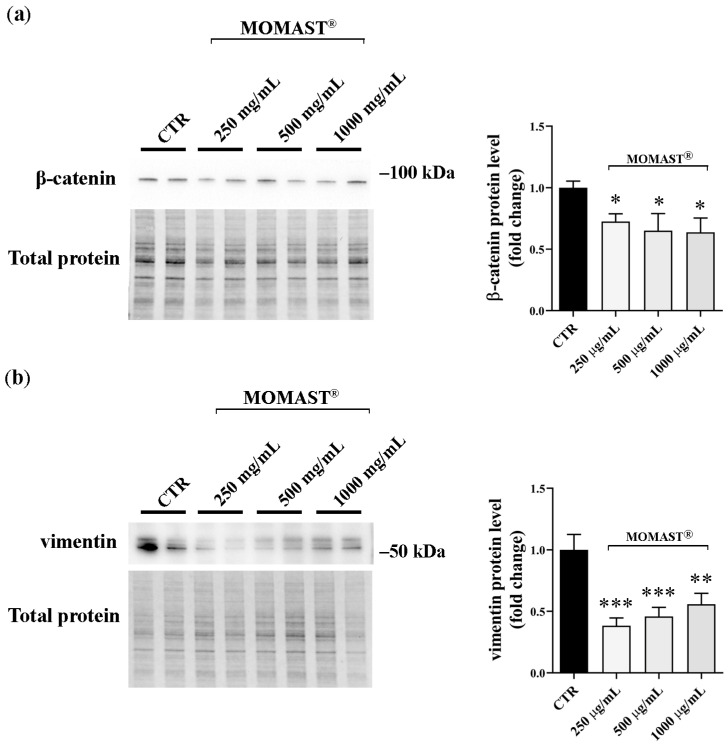
Effect of MOMAST^®^ on EMT markers expression in HCT8 cells. Treatments with MOMAST^®^ at increasing concentrations induced a significant reduction in the EMT proteins expression β-catenin (**a**) and vimentin (**b**) likely suggesting a possible role of MOMAST^®^ in modulating the expression level of selective EMT protein markers. Data are shown as means ± Standard Error Means (S.E.M.) of four independent experiments and analyzed with one-way ANOVA followed by Dunnett’s multiple comparisons test (* *p* < 0.05 vs. CTR; ** *p* < 0.01 vs. CTR; *** *p* < 0.001 vs. CTR).

EMT is a complex mechanism that contributes to cancer development. Marker proteins including vimentin and β-catenin may be useful as prognostic biomarkers. Recent data revealed that the upregulation of AQP3 expression is paralleled with EMT-associated proteins resulting in a poor prognosis of gastric cancer [5]. Therefore, Western Blotting studies were carried out to evaluate the effect of MOMAST^®^ on EMT-related proteins. Cell lysates were immunoblotted using selective antibodies specific to detect vimentin and β-catenin. Obtained data (Figure 9) revealed that treatment with MOMAST^®^ significantly reduced the abundance of vimentin and β-catenin compared to that measured in the lysates isolated from untreated cells. On the other hand, the expression of E-cadherin was not affected by the treatment with MOMAST^®^ (Supplementary Materials, Figure S2).

## 4. Discussion

The major finding of this study indicates that MOMAST^®^ can modulate AQP3 expression and HCT8 cell viability. AQP3 is an aquaglyceroporin mainly expressed in the system of the digestive tract, from the oral cavity to the gastrointestinal tract [29]. In the digestive system, AQP3 is primarily involved in controlling water transport. Abnormal expression of AQP3 has been associated with diarrhea and constipation [30]. However, several studies have suggested that AQP3 plays a role in controlling intestinal barrier integrity [31], cell migration, and proliferation [32]. Recent evidence has shown that AQP3 is involved in cancer progression and metastasis by modulating intracellular signaling that alters cellular responses [32]. In human colon adenocarcinoma HCT8 cells, treatment with DDAVP, a stable analog of the hormone vasopressin, was previously shown to significantly reduce membrane expression of AQP3, resulting in decreased cell viability [6]. Conversely, *Helicobacter pylori* (*H. pylori*) infection, which promotes the generation of proinflammatory interleukins, significantly upregulates AQP3 expression by stimulating HIF-1α and ROS in gastric cells, linking *H. pylori* infection to increased AQP3 expression and gastric cancer development [33]. Here, the action of MOMAST^®^ on AQP3 expression and functions was assayed in HCT8 cells. MOMAST^®^ is a patented natural complex enriched in phenolic molecules and obtained from olive wastewater (OWW) [21]. Several in vitro and in vivo studies have demonstrated that MOMAST^®^ has multiple beneficial effects. In fact, in rat-isolated tissue and different cell models, MOMAST^®^ has anti-inflammatory and antioxidant effects [19,21]. In addition, MOMAST^®^ shows a cholesterol-lowering effect in a hypercholesterolemic mouse model and hepatic HepG2 cells, and in fact it is now commercially available as a sustainable supplement for maintaining the lipid profile balance. Also, several studies have demonstrated that in several cancer cell lines, polyphenols enriched in OWW, including hydroxytyrosol, tyrosol, oleuropein, and verbascoside, prevent inflammation by modulating intracellular oxidative balance and cell viability through apoptosis [34,35,36,37,38]. In the present study, treatment with MOMAST^®^ significantly reduced HCT8 cell viability. Importantly, the concentrations used for this study are within a similar range as the real blood concentration after taking the supplement [18,39]. Moreover, the MOMAST^®^-induced decrease in cell survival was correlated with a significant reduction in AQP3 protein abundance. AQP3 mRNA expression was unaffected. These findings likely suggest that MOMAST^®^ may increase the degradation of the AQP3 protein, which is therefore correlated with decreased cell viability [6]. Functional studies were then performed to assess whether the reduction in AQP3 protein abundance correlated with a decrease in its transport function abilities. By increasing external H_2_O_2_, the fluorescence signal of the HyPer-3 probe increased in parallel. This is consistent with the endogenous expression of peroxiporins in HCT8 cells. Accordingly, acute incubation with DFP00173, a selective inhibitor of AQP3, significantly reduced the H_2_O_2_ transport induced by external H_2_O_2_ at 50 µM. These findings likely indicate that AQP3 contributes to H_2_O_2_ uptake in HCT8 cells. Moreover, exposure to MOMAST^®^ (250 µg/mL) also reduced the fluorescence signal of the HyPer-3 probe induced by external H_2_O_2_ to a similar extent as that observed when cells were incubated with DFP00173. Therefore, a decrease in AQP3 protein abundance and function could lead to increased steady-state levels of H_2_O_2_, making cells more susceptible to oxidative stress, thus compromising cell survival [40]. On the other hand, extracellular H_2_O_2_ may also stimulate, through multiple mechanisms [41], intracellular signals affecting cell viability. It cannot be excluded, and this is a limitation of the study: that the reduction in reactive species induced by MOMAST^®^ may be due to the activation of selective intracellular pathways rather than the direct action of the antioxidants in the extract. In fact, beyond modulating the intracellular H_2_O_2_ balance, MOMAST^®^ significantly reduced the glycerol uptake at the concentration used in this study. Importantly, the uptake of glycerol can contribute to cell growth and viability in two ways: firstly, glycerol is a key substrate necessary for gluconeogenesis, and it is used for the synthesis of triacylglycerols and phospholipids synthesis; secondly, glycerol represents an important modulator for the generation of ATP. Both pathways are necessary for promoting cell growth and proliferation [42,43]. However, it remains unclear whether AQP3-dependent glycerol and/or H_2_O_2_ transport is associated with metabolic disorders and cancer progression. Nevertheless, it is well established that AQP3 is upregulated in different cancers [44,45,46]. Accordingly, selective inhibition of AQP3 reduced cell viability and retarded cancer progression, possibly degrading its transport abilities [6,47,48]. Specific suppression of AQP3 function with DFP00173 decreased HCT8 cell viability and the glycerol rate intake [6]. Interestingly, cells treated with MOMAST^®^ and DFP00173 showed a very low intracellular glycerol transport compared with cells treated with DFP00173 alone. These results indicate that MOMAST^®^ reduced AQP3 protein levels compared to untreated cells. DFP00173, by interacting with the NPA boxes of the AQP3 [49], can further impair the transport ability of the residual AQP3 protein under MOMAST^®^ treatment. The expression and function of AQP3 have been correlated with the stimulation of several signal transduction pathways involved in cell invasion and EMT [32]. The EMT is a complex mechanism involving a number of protein biomarkers including vimentin, E-cadherin, and β-catenin. Furthermore, the activation of several signal transduction pathways, (i.e., Wnt, Notch, EGF) can promote EMT [50]. These findings suggest that each EMT protein can be individually regulated. In gastric cancer, AQP3 induced EMT by modulating the expression of known EMT markers, including vimentin and β-catenin [5,51]. Specifically, vimentin expression is accompanied by increased invasiveness and metastasis formation [42]. Here, vimentin and β-catenin levels were dramatically reduced by treatment with MOMAST^®^. By contrast, no relevant changes in the expression level of E-cadherin were detected. In this respect, it has been also shown that a decrease of E-cadherin expression is indeed not required for the progression of EMT [50]. In the present study, the biological action of MOMAST^®^ was tested. MOMAST^®^ is a complex polyphenolic mixture that contains a variety of molecules that can exert a number of effects, either individually or synergistically. Therefore, in the context of this study, we may speculate that MOMAST^®^ can alter the expression of only selective EMT markers such as vimentin and β-catenin. The main limitation of this study is the use of only HCT8 cells as a unique model to evaluate the effect of MOMAST^®^ on AQP3 expression and function. Further studies, including other in vitro (i.e., HT29 and SW620) and in vivo models, would better clarify and define the action of this olive by-product in order to develop a possible protocol for clinical use and/or food supplementation and assess its broader applicability and physiological relevance

## 5. Conclusions

**Scheme 2:**
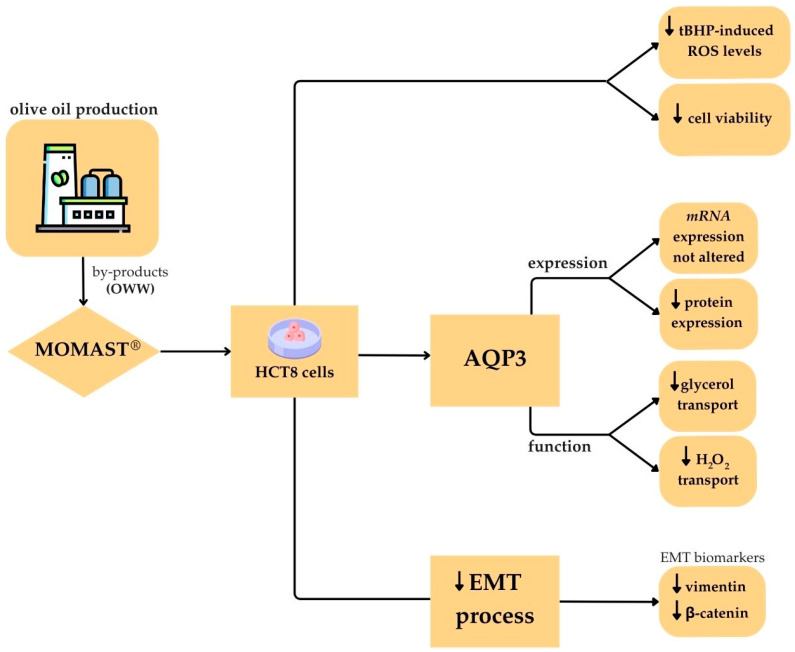
Model depicting the effects of MOMAST^®^ exerted in HCT8 cells. Specifically, MOMAST^®^-reduced AQP3 expression and function and affect the expression level of the EMT protein markers, vimentin and β-catenin. The downward arrow indicates a reduction.

Based on the involvement of AQP3 in the modulation of cell viability and cancer progression through the modulation of the EMT process, these data propose that MOMAST^®^, beyond its antioxidant ability, can modulate other intracellular signaling affecting cell survival. Data are summarized in Scheme 2. However, future studies are needed to clearly define MOMAST^®^ as a potential nutrient supplement useful in diseases characterized by abnormal cell growth by targeting AQP3. 

## Data Availability

All of the data are contained within the article and the Supplementary Materials.

## Supplementary Materials

The following supporting information can be downloaded at: https://www.mdpi.com/article/10.3390/antiox14010026/s1: Table S1. ROS content in HCT8 cells exposed to MOMAST^®^; Figure S1. Effect of MOMAST® on glycerol permeability; Figure S2. Effect of MOMAST^®^ on EMT markers expression in HCT8 cells.
