# Supplementary material for: MOMAST® Downregulates AQP3 Expression and Function in Human Colon Cells
Source: Antioxidants (Basel). 2024 Dec 28;14(1):26. doi: 10.3390/antiox14010026 (PMC11762842; doi:10.3390/antiox14010026)
Supplement: Supplementary file 1 [file antioxidants-14-00026-s001.zip › antioxidants-3363513-supplementary.pdf]

antioxidants

Table S1. ROS content in HCT8 cells exposed to MOMAST®. The table resumes the ROS% content measurements reported as means ± Standard Error Means (S.E.M.) analyzed by one-way ANOVA followed by Dunnett's Multiple Comparison test (**** p<0.0001 vs CTR; ## p<0.01 vs tBHP; ### p<0.001 vs tBHP; #### p<0.0001 vs tBHP).

	**mean ± S.E.M.**
**CTR**	1,00 ± 0,08
**tBHP**	13,58 ± 1,83****
**MOMAST® 50 μg/mL**	1,43 ± 0,12
**MOMAST® 100 μg/mL**	1,34 ± 0,21
**MOMAST® 250 μg/mL**	1,13 ± 0,09
**MOMAST® 500 μg/mL**	1,14 ± 0,12
**MOMAST® 1000 μg/mL**	1,03 ± 0,07
**MOMAST® 50 μg/mL + tBHP**	10,96 ± 0,88
**MOMAST® 100 μg/mL + tBHP**	8,84 ± 1,05###
**MOMAST® 250 μg/mL + tBHP**	9,64 ± 0,97##
**MOMAST® 500 μg/mL + tBHP**	9,47 ± 0,96##
**MOMAST® 1000 μg/mL + tBHP**	7,44 ± 0,68####

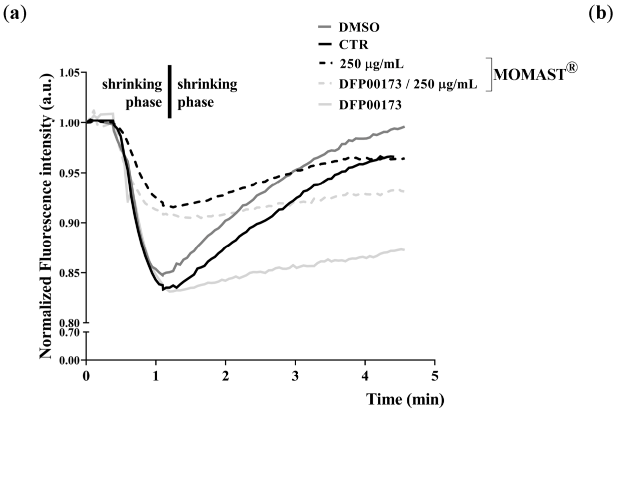

Figure S1. Effect of MOMAST® on glycerol permeability. Osmotically induced volume changes were recorded by the calcein-quenching method in HCT8 cells treated with MOMAST® compared to the control condition (untreated). (**a**) Representative time courses of cell shrinking (water exit) followed by cell swelling indicative of the osmotic influx of water promoted by glycerol entry along its gradient (Δ 100 mOsm/L). (**b**) Histogram showing the means ± Standard Error Means (S.E.M.) values of the cell swelling time constants (slope) reflecting glycerol entry into the cells. Data are obtained from 91 different measurements of 3 independent experiments. A one-way ANOVA and Dunnett's Multiple Comparison test were performed (**** p<0.0001 vs CTR; #### p<0.0001 vs DMSO).

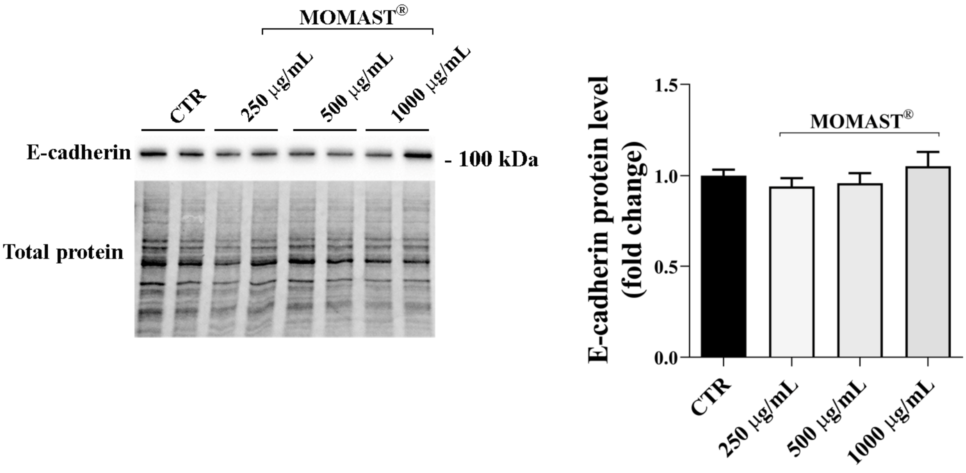

Figure S2. Effect of MOMAST® on EMT markers expression in HCT8 cells. Treatments with MOMAST® at increasing concentrations did not alter the E-cadherin adhesion protein expression. Data are shown as means ± Standard Error Means (S.E.M.) of 4 independent experiments and analyzed by one-way ANOVA followed by Dunnett's multiple comparisons test.
